# Effects of Scopoletin Supplementation and Stocking Density on Growth Performance, Antioxidant Activity, and Meat Quality of Korean Native Broiler Chickens

**DOI:** 10.3390/foods10071505

**Published:** 2021-06-29

**Authors:** Sang Hun Ha, Hwan Ku Kang, Abdolreza Hosseindoust, Jun Young Mun, Joseph Moturi, Habeeb Tajudeen, Hwa Lee, Eun Ju Cheong, Jin Soo Kim

**Affiliations:** 1 Department of Bio-Health Convergence, Kangwon National University, Chuncheon 24341, Korea; kayne7602@kangwon.ac.kr (S.H.H.); hosseindoust@kangwon.ac.kr (A.H.); 202016455@kangwon.ac.kr (J.Y.M.); motkondo@gmail.com (J.M.); habeebtop@gmail.com (H.T.); 2 Poultry Research Institute, National Institute of Animal Science, Pyeongchang 25342, Korea; magic100@korea.kr; 3 Department of Animal Industry Convergence, Kangwon National University, Chuncheon 24341, Korea; 4 Department of Forest Environmental System, Kangwon National University, Chuncheon 24341, Korea; dlghk018@kangwon.ac.kr

**Keywords:** broilers, stress, welfare, corticosterone, productivity

## Abstract

Stocking density stress is one of the most common management stressors in the poultry industry. The present study was designed to investigate the effect of dietary *Sophora koreensis* (SK; 0 and 20 mg/kg diet) and stocking density (SD; 14 and 16 chickens/m^2^) on the antioxidant status, meat quality, and growth performance of native Korean chickens. There was a lower concentration of malondialdehyde (MDA) and a higher concentration of catalase, superoxide dismutase (SOD), and total antioxidant capacity in the serum and leg muscle with the supplementation of SK. The concentration of MDA was increased and concentrations of SOD were decreased in the leg muscle of chickens in low SD treatments. The SK-supplemented treatments showed an increased 3-ethylbenzothiazoline-6-sulfonate-reducing activity of leg muscles. The higher water holding capacity of breast muscle and a lower cooking loss and pH were shown in the SK-supplemented treatments. The addition of dietary SK resulted in a greater body weight gain and greater spleen and bursa Fabricius weight, as well as lower feed intake and abdominal fat. The low SD and supplementation of SK increased the concentrations of cholesterol. The concentration of glucose was increased in the low SD treatment. Corticosterone level was decreased in the SK-supplemented and low SD treatments. In conclusion, SK supplementation reduced the oxidative stress and increased meat quality and antioxidant status of chickens apart from the SD stress.

## 1. Introduction

Stocking density (SD) exceeding the comfort zone causes stress in farm animals [1]. Presently, the SD ratio is markedly increasing worldwide to minimize costs. The continuous increase in the SD of broiler chickens in the poultry industry to decrease production costs increases health and welfare issues [2]. In addition to low body weight gain, production of broiler chickens in a high-SD situation decreases meat quality including water-holding capacity and meat tenderness by increasing the oxidative reactions [1,3]. As the traditional broiler’s meat is costly because of higher meat quality compared with modern broilers, the low meat quality can compromise the marketability. Moreover, the high stress level affects polyunsaturated fatty acids of meat, which increases the vulnerability to oxidative deterioration [3,4,5]. The control of lipid oxidation associated with SD entails the supplementation of antioxidant factors to block the production of free radicals [6]. Several important macromolecules or enzymes are under the influence of reactive oxygen species (ROS) and free radicals, which have the potential of increasing lipid peroxidation in organs [7,8,9]. An oxidative stress condition in animals refers to the progressive loss of anti-oxidative status caused by various internal stressors or environmental factors. Normally, the natural antioxidant defense system is able to prevent cells from oxidative injuries by enzymatic control of free radicals [10]. Superoxide dismutase (SOD), glutathione peroxidase (GPx), and catalase are the major enzymes to counteract free radicals and diminish the lipid peroxidation rates [11,12]. In addition, the lower antioxidant defense in the body results in the excessive generation of ROS leading to oxidative injuries [6]. Therefore, improvement of oxidative status may alleviate the detrimental effects of high SD on growth performance and meat quality.

*Sophora koreensis* (SK), from the Fabaceae family, is a perennial herb in the mountainous area of the Korean Peninsula [13]. The root and flower of SK species contain a large quantity of flavonoids [14], isoflavonoids [15], and scopoletin [16,17] having anti-oxidative capacity [18]. Scopoletin is a kind of phenolic coumarin with promising anti-inflammatory effects [19] that can protect the body from microbial attack and environmental stress, including mechanical injury [20]. The antioxidant properties of scopoletin were proved by scavenging superoxide anion, which may prevent stressful conditions related to oxidative damage [16]. In our dose-dependent pre-study, the antioxidant properties of SK in chickens had been proven (unpublished). Therefore, we hypothesized that the effects of SK can be better highlighted when used during a stressful condition. To our knowledge, there is a lack of reports regarding the antioxidant effects of scopoletin-rich diets, not only on the growth performance but also on the meat quality of broiler chickens during high SD. This experiment was thus designed to determine the effects of a supplementary scopoletin-rich feed additive on meat quality and antioxidant status in broilers under high SD stress.

## 2. Materials and Methods

### 2.1. Experimental Design, Chickens, and Diets

The experiment was approved by the Institutional Animal Care and Use Committee, Kangwon National University (KW-170519-1). Three hundred and twelve Korean native chickens (Hanhyup 3, 914.3 ± 26.3 g, 49 days old) were fed for 35 days. A scopoletin-rich product extracted from SK was added to the experimental diets from the first day of study. Four treatments included SK supplementation levels (0 and 20 ppm) and stocking densities (14 and 16 bird/m^2^). Each treatment consisted of 6 replicates with 13 birds per replicate (floor pen, w2350 × d1500 × h850 mm), where the rice hull was used as litter. The temperature and humidity of the broiler house were controlled by an automatic ventilation system to have an average temperature and humidity of 22.1 °C and 46%, respectively. The diet contained 18% crude protein, 3100 kcal/kg metabolizable energy, 0.86% calcium, 0.33% available phosphorus, 0.21% sodium, 1.01% available lysine, 3.5% crude fiber, and 6.86% ether extract. White light was provided (25 lux at bird-head level), with a light schedule of 19L:5D (lights off from 2200 to 0300 h), and water and feed were maintained ad libitum. The mash-type diet was prepared to meet the nutrient requirements of Korean native chickens. The SK supplement contained 2090.5 mg scopoletin/kg (Table 1).

### 2.2. Sample Collection

At day 35, a total of 72 birds, 18 chickens per treatment (3 chickens/replicate), were used for carcass characteristics analysis, meat quality, relative organ weight, antioxidant status, and plasma metabolites evaluation. Birds in the same range of bodyweight (BW) based on the average BW of a treatment were applied. Blood samples were taken from the wing vein by using a syringe. Collected blood was moved into non-treated vacuum tubes, and was immediately sent for plasma separation, then centrifuged at 2500× *g* for 10 min. Serum was aspirated and located in a 2.5 mL centrifuge tube then stored at −20 °C before analysis for malondialdehyde (MDA), catalase, SOD, and total antioxidant capacity (TAC). All selected birds were decapitated at the first cervical vertebrae. After defeathering and removal of organs and feet, the carcass, breast muscles (both sides), drumsticks (both sides), and abdominal fat were weighed and then stored at −20 °C. The meat quality, antioxidant status, and weights of liver, spleen, and bursa of Fabricius were measured to calculate the relative carcass weight and internal organ weight, and then these parts were stored at −80 °C.

### 2.3. Antioxidant Status in Serum and Muscle

In order to assay the antioxidant factors activity in muscle and serum, the samples were pre-treated and measured as explained by Hosseindoust et al. [5] by using a Cayman kit manual (Enzyme activity assay, Cayman Chemical, Ann Arbor, MI, USA). The harvested samples from the muscle and serum were subjected to the evaluation of the concentration of MDA (Cat #10006438, Cayman Chemical, Ann Arbor, MI, USA), catalase (Cat #707002, Cayman Chemical, Ann Arbor, MI, USA), SOD (Cat #706002, Cayman Chemical, Ann Arbor, MI, USA), and TAC (Cat #709001, Cayman Chemical, Ann Arbor, MI, USA). To evaluate the absorption detection, a microplate reader (Power Wave XS, BIoTeK, Winooski, VT, USA) was applied according to the Cayman kit’s manufacturer’s manual.

### 2.4. Radical Scavenging Capacity

The evaluation of 3-ethylbenzothiazoline-6-sulfonate (ABTS)-reducing activity was performed using the supernatant collected from thigh meat according to Hosseindoust [5] and Blois [21]. In brief, 200 μL of supernatant was placed in a 5 mL centrifuge tube and added to 800 μL deionized distilled water. The determination of ABTS-reducing activity and the preparation of the ABTS solution were conducted following the method described by Erel [22]. The prepared ABTS solution was diluted with ethanol for adjusting absorbance approximately 0.70 at 734 by using a UV spectrophotometer (Optizen 3220UV, MECASYS, Daejeon, Korea). The diluted ABTS solution (3 mL) was mixed with 20 μL of supernatant and the absorbance was measured by a UV spectrophotometer at 734 nm. The ethanol was used as a blank. The percentage inhibition was obtained by the following equation: ABTS-reducing activity (%) = ((absorbance of the control − the absorbance of the sample)/absorbance of the control) × 100.

### 2.5. Meat Quality

Leg muscle (with bone, and without skin, tendon, and fat) meat color was examined using a Chroma Meter CR-400 instrument (Minolta Co., Osaka, Japan) according to International Commission on Illumination (CIE) L * (lightness), a * (redness), and b * (yellowness). Water holding capacity (WHC), cooking loss (%), shear force (%), pH, and MDA were examined using muscle pectoralis major according to Hosseindoust et al. [5]. The WHC was measured by placing a 0.5 g meat sample on a round plastic plate in a tube (Millipore Ultrafree-MC; Millipore, Bedford, MA). The samples were heated for 20 min (80 °C) in a water bath, then cooled (23 ± 1 °C), and centrifuged (2000× *g*) for 10 min (4 °C) to evaluate WHC as follows: WHC = (moisture content−water loss)/moisture content × 100. To measure the cooking loss, 3 g of leg muscle meat was placed in a plastic bag and heated in a water bath (85 °C) for 20 min, then cooled at room temperature to calculate cooking loss according to this equation: (sample weight before cooking/sample weight after cooking)/sample weight before cooking × 100 [5]. For shear force determination, a texture analyzer (TA-XT2i, Stable Microsystems, Surrey, UK) equipped with a Warner-Bratzler shear blade, a 25 kg load cell, and a test speed setting at 2.0 mm/s was used with the maximum force (kg) [23]. The pH of meat was evaluated as explained by Hosseindoust et al. [5]. In brief, a 5 g sample of meat was homogenized in distilled water (45 mL) by a homogenizer (DIAX 900, Heidolph, Kelheim, Germany) for 15 s, and then the pH was determined by using a pH meter (Orion 230A Thermo Fisher Scientific, Waltham, MA, USA). After performing the pH process, a Watman no. 2 (Hillsboro, OH, USA) was used to filter homogenized samples.

### 2.6. Blood Metabolites

The mixed blood samples with K_2_ EDTA were used for determination of total cholesterol, total protein, triglyceride, glucose, glutamic pyruvic transaminase (GPT), glutamate oxaloacetate transaminase (GOT), albumin, phosphate, and calcium (Hemavet 950, Drew Scientific, Miami Lakes, FL, USA). The serum corticosterone was analyzed using the ELISA kit (Corticosterone ELISA kit, Enzo life Sciences, Farmingdale, NY, USA).

### 2.7. Statistical Analysis

The experimental values were analyzed by GLM procedure of SAS^®^ 9.3 software (SAS Inst. Inc., Cary, NC, USA). The pen was used as the experimental unit for the analysis of growth performance, and an individual chicken was considered as the experimental unit for measuring the blood, meat quality, and carcass trait samplings. The difference of means was tested by Tukey test. The effect of dietary SK supplementation and stocking densities and their interactions were determined. A significant difference was expressed either *p* < 0.01 or *p* < 0.05.

## 3. Results

### 3.1. Antioxidant Factors

There was a significantly lower (*p* < 0.01) concentration of MDA in the serum with the supplementation of SK (Table 2). The serum concentrations of catalase (*p* < 0.05) and SOD (*p* < 0.05) were increased by supplementation of SK in the diet, while there was no difference between the SD treatments. The addition of dietary SK resulted in a greater (*p* < 0.01) TAC in the serum; however, the SD treatments did not change the TAC capacity. The low SD (*p* < 0.05) and supplementation of SK (*p* < 0.01) decreased the content of MDA in the leg muscle. The concentrations of catalase (*p* < 0.01) and SOD (*p* < 0.05) were increased by supplementation of SK in the diet, while there was a decrease in the concentration of SOD in the high SD treatment. There was an increased (*p* < 0.01) TAC in the leg muscle of chickens with increased supplementation of SK; however, there were no significant effects of SD on TAC in the leg muscle.

### 3.2. ABTS-Reducing Activity

**Figure 1:**
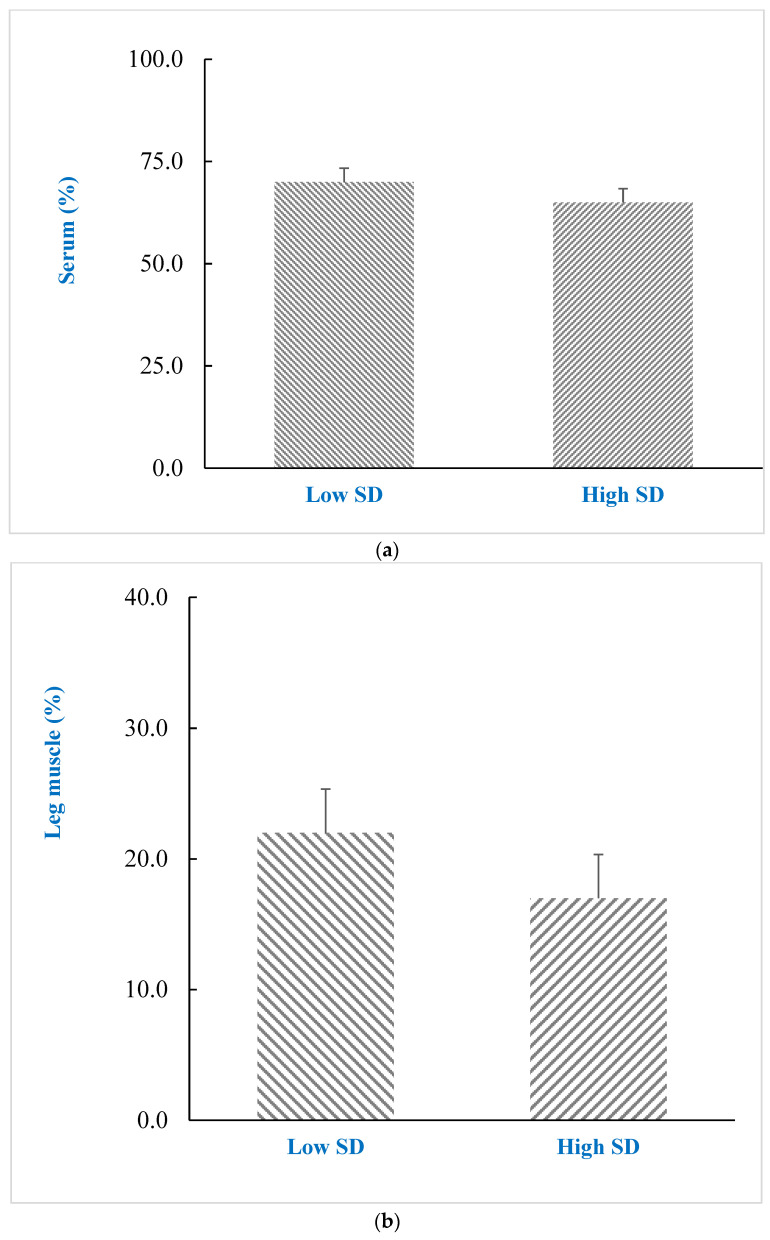

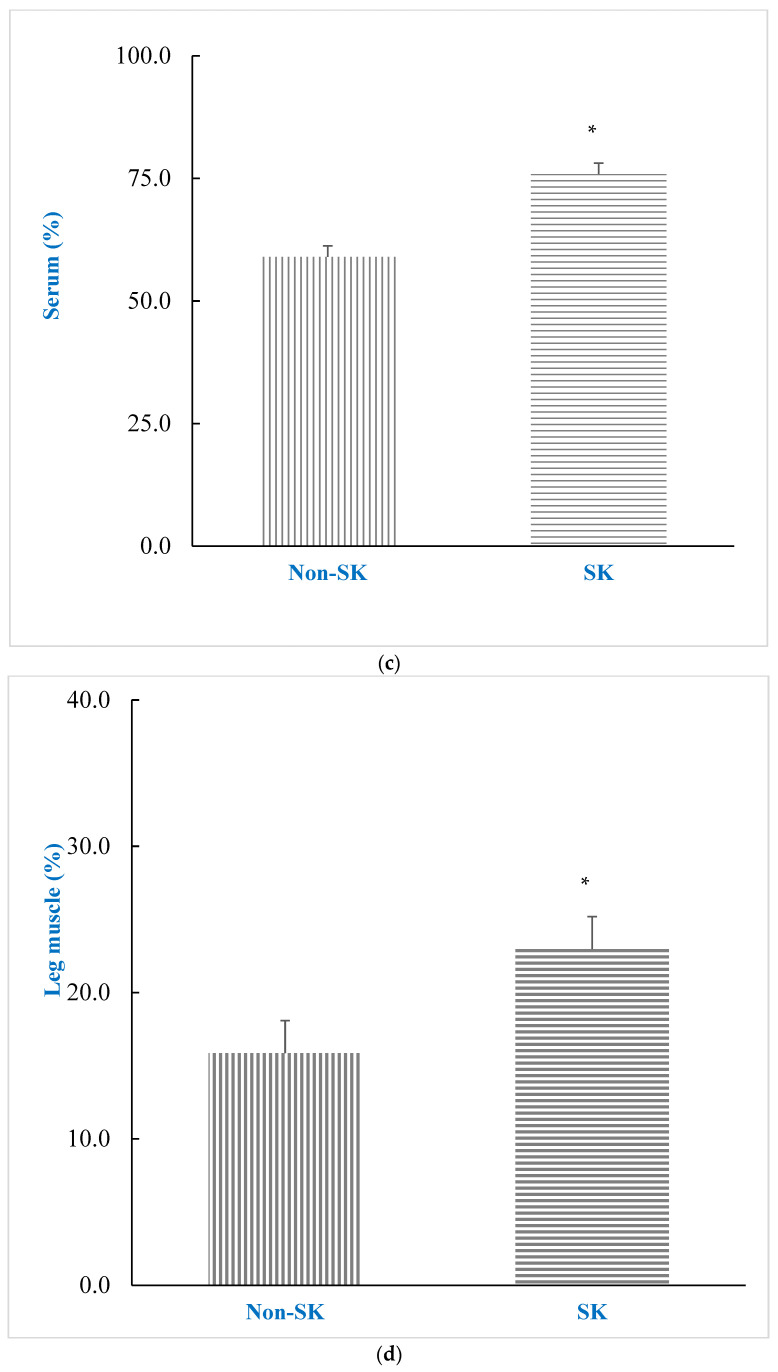
ABTS radical scavenging capacity (%) of different stocking density (SD) on serum (**a**) and leg muscle (**b**), and *Sophora koreensis* (SK) supplementation on serum (**c**) and leg muscle (**d**) of Korean native chicken. Non-SK, basal diet; SK, basal diet + 20 ppm SK; Asterisks (*) indicate statistical significance (*p* < 0.05).

The antioxidant capacity result of serum indicated that ABTS-reducing activity was enhanced in the SK-supplemented treatments (Figure 1). No difference in ABTS-reducing activity was detected between the SD treatments. The SK-supplemented treatments showed an increased (*p* < 0.05) ABTS-reducing activity of leg muscles compared with the non-SK-supplemented treatments; however, there was a greater ABTS-reducing activity of leg muscle in the SK-supplemented treatments.

### 3.3. Meat Color and Meat Quality

The effect of diets and SD on breast meat color and quality of chickens was shown in Table 3. There were no breast meat redness, lightness, and yellowness responses to supplementation of SK in the diet and rearing in different stocking densities. The higher water holding capacity of breast muscle was shown for the SK-supplemented treatments (*p* < 0.01); however, there was no change among the SD treatments. A lower cooking loss and breast muscle pH was reported in the SK-supplemented treatments (*p* < 0.01), although there were no changes in cooking loss and pH of breast muscle between the SD treatments. The shear force was not affected by treatments.

### 3.4. Growth Response, Carcass Traits, Immune Organ Ratio

Table 4 shows the influences of diets and SD on growth performance, carcass traits, and relative organ weight. The effect of SK supplementation on improving final BW, BW gain, and feed intake of chickens was significant (*p* < 0.01). There was no difference in feed conversion ratio between the SK treatments. There was no difference in final BW and BW gain between the SD treatments; however, the feed intake and feed conversion ratio of chickens were adversely affected by the high SD. The carcass yield, breast meat, and drumsticks showed no dietary SK effects; however, a lower (*p* < 0.01) abdominal fat was shown in the SK-supplemented treatments. The carcass yield, breast meat, drumstick percentage, and abdominal fat were decreased in the high SD treatments. There was no change in the relative weight of the liver. There were significant interactions between the SD and SK in increasing the relative weight of the spleen and bursa Fabricius. The main effects shown were that the relative weight of the spleen and bursa Fabricius were increased by supplementation of SK, but decreased by increasing the SD. There were no differences in the percentage of the spleen to bursa Fabricius and relative weight of thyroid between the treatments.

### 3.5. Blood Profile

The effects of dietary SK on blood profile are shown in Table 5. Results indicated that total cholesterol and glucose levels were decreased in the high SD treatment but that the concentration of blood total cholesterol was increased in the SK-supplemented treatments. There was no change in concentration of total protein, triglyceride, GPT, GOT, albumin, phosphate, and calcium among the treatments. The blood corticosterone level was significantly higher (*p* < 0.05) in the high SD treatment; however, corticosterone level was decreased in the SK-supplemented treatments.

## 4. Discussion

*Sophora koreensis* has been known as a traditional herb to treat rheumatoid issues in Korea because of its antioxidant capacity [13,24]. The antioxidant enzymes including SOD, GPx, and catalase are the first factors against antioxidant reactions [11,12]. Antioxidant effects of scopoletin were shown earlier [16,17]. An increase in the aforementioned enzyme’s production capacity can improve the antioxidant system by controlling the production of ROS. Superoxide dismutase is an important enzyme in the protection of cells from adverse effects of ROS [25]. In the current study, decreased concentration of MDA and increased TAC in the serum of chickens treated with SK may be related to the increased concentrations of catalase and SOD, which reduce the formation of peroxides and hydroperoxides in fat tissues [7]. The increased activities of catalase and SOD in the serum of broiler chickens fed SK diets show its potential to scavenge free radicals during the stressful condition of high SD. It has been reported that the stressful condition decreases the SOD and catalase production, which in turn increases MDA production [26]. There is a positive correlation between high MDA concentrations and lipid peroxidation [7,8]. Meanwhile, the reduction of lipid peroxidation in the thigh muscle may be reflected in the decrease of MDA concentration in the SK treatments. Scopoletin also showed high inhibitory activities against anti-inflammatory cytokines by decreasing TNF-α, IL-1β, and IL-6 secretions [17,19,27]. It has been reported that the anti-inflammatory influences of scopoletin are associated with a decrease in free radicals production. The production of MDA, as an important indicator of lipid peroxidation, is due to the exposure of free radicals to the plasma membrane [7,8,28]. Lee et al. [29] reported that the MDA levels were significantly increased in mice under alcoholic food stress, but that the supplementation of scopoletin prevented the increase in MDA concentration compared with the control treatment. Several challenge experiments with high inflammatory condition confirmed that the decrease in inflammation mediates cell damage, lipid peroxidation, and increases the inactivation of antioxidant enzymes [9,10,28]. Several researchers have reported that high SD could increase the stress level and decrease the antioxidant status by increasing MDA and decreasing SOD concentrations in serum [1,2,30]. Although increased catalase and TAC seem to be consistent in the plasma and leg muscle in the SK-supplemented treatments, this trend is missing for chickens in the SD treatments. However, chickens in the SD treatments showed a higher MDA and lower SOD content in the leg muscle. Although the decrease in the SOD concentration was in line with the increased MDA in the leg muscle, the concentration of the aforementioned parameters in the serum was not in agreement with the leg muscle results.

Although scopoletin increases the antioxidant status [17], literature did not study the effects of supplemental scopoletin on meat quality related to SD stress in chickens focusing on the antioxidant capacity. Therefore, this study aimed to test the influences of scopoletin on the antioxidant capacity of meat, plasma, and the possible interactions with the quality and color of meat. Although the scavenging capacity of scopoletin against ABTS in chicken meat has not been studied, an in vitro study on the antioxidant role of phenolic compounds reported that the α-diphenyl-β-picrylhydrazyl radical-scavenging activity of scopoletin was around 11,800 times higher than vitamin C, making it a potent antioxidant compound [31]. The increased scavenging capacity of ABTS of meat due to scopoletin was in line with an enhanced TAC in the leg muscle, indicating the reduction of oxidative damage in muscle tissues with the presence of scopoletin. Furthermore, an increased SOD, as well as decreased MDA, content in the serum and meat confirm the capability of scopoletin in decreasing oxidative stress. Therefore, the result of the current study showed that the supplementation of 20 ppm scopoletin adequately increased the ABTS-reducing activity in chickens.

In the present study, the abdominal fat percentage of chicken was shown to be lower in the SK-supplemented group than in the non-supplemented group, which was in line with the results reported by Rajaei et al. [32], who reported that the addition of 5 mL/L of noni juice, as a rich source of scopoletin, in drinking water had a significant effect in decreasing abdominal fat content in broiler chickens. Scopoletin is known as a stimulator of fatty acid oxidative genes including PPARa, Acsl1, CPT, Acox, and Acaa1a, and an inhibitor of lipogenic genes such as sterol regulatory element-binding protein-1c and fatty acid synthase in the white adipose tissue and liver in rat [29,33]. The reduced abdominal fat may be due to the supplementation of scopoletin as a bioactive component that reduces oxidative stress and improves carbohydrate and fat metabolism. Serum corticosterone can reflect the welfare status of chickens with the environment [34], and several production and behavioral parameters can be under the influence of corticosterone hormone. Hosseindoust et al. [5] reported that stress hormone is a decisive index for deposition of protein and carcass percentage in chickens. Supplementation with *Morinda citrifolia* L. as a rich source of scopoletin increased the absorption of amino acids, which subsequently increased the carcass rate of broiler chickens [32]. In the current study, the decrease of abdominal fat in the SK-supplemented treatments may not be due to the serum corticosterone because of the insignificant difference in breast meat and drumstick. However, chickens in the SD treatments showed a lower breast meat and drumstick percentage as well. The lower protein deposition may decrease the percentage of muscle to fat and be responsible for the higher relative abdominal fat. There is a positive correlation between corticosterone concentration and abdominal, thigh, or cervical adipose tissue’s fat contents [35]; however, the degradation of skeletal muscle can be increased when the serum corticosterone concentration increases [36]. Therefore, the lower protein deposition in muscular organs including breast muscle and drumstick may be responsible for the higher relative abdominal fat.

The health status and BW of animals are under the adverse effect of SD stress, which may cause economic loss [1,2]. Corticosterone is one of the most common end-products of stress and will be secreted to the blood during stressful environments [5,34,37]. Long-time exposure to restraints disrupts the hypothalamic-pituitary-adrenal axis and increases the concentrations of plasma corticosterone [38]. Consistently, the current study showed that the supplementation of SK in the diet during SD stress led to the decrease of corticosterone in the serum. The excessive production of ROS compromises cell growth by degrading cytoskeletal proteins, as well as causing lipids peroxidation during stressful conditions [9]. Our result is in line with those from other researchers who have employed SD stress in poultry [34].

Our study showed that dietary SK supplementation improved the meat quality of chickens by increasing water holding capacity and decreasing cooking loss. The water holding capacity is known as a determinantal factor of meat quality [3]. The significantly lower cooking loss in SK-supplemented treatments may be due to the lower serum corticosterone level. It was reported that excessive levels of serum corticosterone not only reduced the BW gain through reducing anabolism and increasing catabolism processes [39], but also induced lipid peroxidation [40,41], which may influence meat quality. The protective roles of antioxidant enzymes and controlling the effects of free radical damage seem essential to reduce the adverse influences of stocking density stress. Antioxidant properties of scopoletin may be reflected in the MDA concentration of meat. The lower concentration of serum corticosterone may indicate that the chickens fed SK diets had relatively lower stress regardless of SD because the SD did not decrease cooking loss of breast meat. There is a positive relationship between corticosterone production and the secretion of inflammatory cytokines including IL-1b, IL-6, IL-10, IL-12-a, and IL-18 [19,37], which can adversely affect meat quality. Corticosterone secretion has been shown to be a factor to decrease meat quality by degrading protein in muscle and decreasing the fatty acid transport protein expression [35]. Song et al. [42] stated that the concentration of uric acid, as a factor to show protein catabolism in tissues, increased in blood when the concentration of corticosterone increased in the blood. In addition, the production of ROS can be increased by corticosteroid hormones [43]. The high antioxidant status leads to lower exposure to hydroxyl and peroxyl radicals and possibly the protection of lipid tissues from oxidation through chelating free radicals [4,6]. The result of radical scavenging capacity in this study shows that scopoletin is a potent antioxidant factor to protect fatty acids oxidation and increase meat quality.

The BW gain of chickens in the high SD treatments was 11.9% lower than the low SD treatments. In addition, feed intake was 13.9% lower in the high SD treatments. The reduction of weight gain and feed intake may show that the SD stress adversely affected the performance of broiler chickens. There are important biomarkers such as corticosterone that decrease growth performance during a stressful period [34,38]. The greater BWG in SK-supplemented groups may be because of the antioxidant effects of scopoletin, which may stimulate protein synthesis.

## 5. Conclusions

In conclusion, to improve radical scavenging capacity and controlling lipids peroxidation, the antioxidant capacity of broiler chickens can be improved by SK supplementation during high SD stress. Dietary SK improved meat quality through increasing ABTS radical scavenging capacity in the serum and leg muscle. In addition, lower abdominal fat and higher immune organs weight were shown in chickens fed SK. Therefore, our study suggests that SK is a practically useful feed additive to improve the meat quality and weight gain of chickens regardless of SD stress.

## Figures and Tables

**Table 1:** Analyzed composition of *Sophora koreensis*.

Item	*Sophora koreensis*
Scopoletin (mg/kg)	2090.5
Dry matter %	93.12
Crude protein %	12.82
Ether extract %	1.72
Crude fiber %	29.69
Ash %	3.15
Calcium %	1.12
Phosphorus %	0.19
Amino Acids %	
Arg	3.21
His	0.61
Ile	9.58
Leu	23.03
Lys	11.16
Met	5.57
Phe	4.97
Thr	3.03
Trp	1.61
Val	1.06
Fatty acids %	
Palmitic acid	0.43
Oleic acid	1.06
Linoleic acid	0.35
Linolenic acid	0.59
Arachidonic acid	5.18

**Table 2:** Effect of dietary SK and SD on antioxidant activity of serum and leg muscle in Korean native chicken.

Stocking Density (*n*/m^2^)	14		16		SEM	*p*-Values		
*Sophora koreensis* (ppm)	0	20	0	20		SD	SK	SD × SK
Serum								
MDA (nmol/mL)	10.55	5.87	11.41	6.22	0.55	0.584	<0.001	0.818
Catalase (nmol/min/mL)	0.23	0.31	0.21	0.31	0.02	0.844	0.027	0.903
SOD (U/mL)	44.73	61.63	49.33	63.38	0.77	0.053	<0.001	0.369
TAC (mM)	0.15	0.32	0.14	0.30	0.02	0.675	<0.001	0.957
Leg muscle								
MDA (nmol/mg)	0.59	0.51	0.66	0.57	0.01	0.029	0.004	0.794
Catalase (nmol/min/mg)	0.21	0.43	0.21	0.39	0.02	0.685	<0.001	0.591
SOD (U/mg)	39.97	68.78	32.30	50.85	1.53	<0.001	<0.001	0.109
TAC (mM)	0.13	0.37	0.11	0.33	0.01	0.184	<0.001	0.466

SEM, standard error of means; SD, stocking density effect; SK, *Sophora koreensis* supplementation effect; SD × SK, stocking density × *Sophora koreensis* supplementation effect interaction; MDA, malondialdehyde; SOD, Superoxide dismutase; TAC, total antioxidant capacity.

**Table 3:** Effect of dietary SK and SD on breast meat color and quality in Korean native chicken.

Stocking Density (*n*/m^2^)	14		16		SEM	*p*-Values		
*Sophora koreensis* (ppm)	0	20	0	20		SD	SK	SD × SK
Meat color								
Lightness (L *)	53.03	52.92	52.81	52.88	0.32	0.837	0.977	0.882
Redness (a*)	4.14	4.56	4.20	4.45	0.17	0.943	0.350	0.812
Yellowness (b*)	8.18	8.49	8.37	8.61	0.21	0.727	0.525	0.937
Meat quality								
Water holding capacity (%)	44.95	48.27	40.91	47.75	0.62	0.081	0.001	0.172
Cooking loss (%)	30.09	26.81	29.82	25.26	0.61	0.461	0.004	0.602
Shear force (*n*/cm^2^)	23.63	24.91	22.95	24.12	0.39	0.360	0.110	0.965
pH	5.68	5.80	5.81	5.58	0.02	0.132	<0.001	0.089

SEM, standard error of means; SD, stocking density effect; SK, *Sophora koreensis* supplementation effect; SD × SK, stocking density × *Sophora koreensis* supplementation effect interaction.

**Table 4:** Effect of dietary SK and SD on growth performance, carcass traits, and relative weights of organs in Korean native chicken.

Stocking Density (*n*/m^2^)	14		16		SEM	*p*-Values		
*Sophora koreensis* (ppm)	0	20	0	20		SD	SK	SD × SK
Growth Performance								
Final BW (g/bird)	2319	2313	2149	2191	8.12	0.273	<0.001	0.153
BW gain (g/bird)	1408	1399	1231	1276	10.01	0.373	<0.001	0.199
FI (g/bird)	3949	3849	3458	3390	18.29	0.032	<0.001	0.667
FCR (g/bird)	2.81	2.75	2.82	2.66	0.02	0.037	0.356	0.276
Carcass traits (%)								
Carcass yield	71.05	71.56	69.41	69.82	0.20	<0.001	0.269	0.907
Breast meat	19.72	19.96	17.93	18.32	0.13	<0.001	0.228	0.770
Drumsticks	14.17	14.34	13.21	13.34	0.15	0.003	0.622	0.939
Abdominal fat	1.84	1.42	1.66	1.35	0.02	0.009	<0.001	0.211
Relative weights of organs (%)								
Liver	2.67	2.56	2.43	2.41	0.07	0.147	0.623	0.732
Spleen	0.088	0.114	0.079	0.083	0.01	<0.001	<0.001	0.002
Bursa of Fabricius	0.092	0.125	0.087	0.095	0.01	<0.001	<0.001	<0.001
Spleen/bursa	0.923	0.872	0.878	0.847	0.06	0.257	0.184	0.730
Thyroid	0.629	0.655	0.728	0.503	0.03	0.697	0.153	0.078

SEM, standard error of means; SD, stocking density effect; SK, *Sophora koreensis* supplementation effect; SD × SK, stocking density × *Sophora koreensis* supplementation effect interaction; BW, bodyweight; FI, feed intake; FCR, feed conversion ratio.

**Table 5:** Effect of dietary SK and SD on blood profile in Korean native chicken.

Stocking Density (*n*/m^2^)	14		16		SEM	*p*-Values		
*Sophora koreensis* (ppm)	0	20	0	20		SD	SK	SD × SK
Total cholesterol (mg/dL)	106.3	115.6	102.7	105.8	1.49	0.036	0.050	0.302
Total protein (mg/dL)	2.88	2.74	2.73	2.70	0.03	0.144	0.206	0.373
Triglyceride (mg/dL)	55.89	54.09	51.37	54.20	1.62	0.503	0.875	0.482
Glucose (mg/dL)	256.1	254.0	243.1	239.5	2.82	0.024	0.619	0.894
GPT (U/L)	2.07	2.12	2.15	2.19	0.08	0.636	0.778	0.996
GOT (U/L)	214.3	225.7	210.9	217.8	3.22	0.388	0.172	0.728
Albumin (mg/dL)	1.12	1.03	1.10	1.09	0.01	0.568	0.097	0.150
Phosphate (μM/L)	10.14	10.18	10.15	10.02	0.12	0.758	0.850	0.738
Calcium (μM/L)	9.09	9.07	9.10	9.23	0.10	0.686	0.785	0.717
Corticosterone (ng/mL)	53.37	48.42	61.51	51.06	1.05	0.019	0.002	0.206

SEM, standard error of means; SD, stocking density effect; SK, *Sophora koreensis* supplementation effect; SD × SK, stocking density × *Sophora koreensis* supplementation effect interaction; GPT, glutamic pyruvic transaminase; GOT, glutamate oxaloacetate transaminase.
